# Systematic identification and functional characterization of the CFEM proteins in poplar fungus *Marssonina brunnea*

**DOI:** 10.3389/fcimb.2022.1045615

**Published:** 2022-11-10

**Authors:** Yulin Qian, Xinyue Zheng, Xueying Wang, Jun Yang, Xiangyang Zheng, Qirui Zeng, Jinwen Li, Qiang Zhuge, Qin Xiong

**Affiliations:** Co-Innovation Center for Sustainable Forestry in Southern China, College of Biology and the Environment, Nanjing Forestry University, Nanjing, China

**Keywords:** CFEM domain, effector, *Marssonina brunnea*, poplar, plant immunity

## Abstract

Proteins containing Common in Fungal Extracellular Membrane (CFEM) domains uniquely exist in fungi and play significant roles in their whole life history. In this study, a total of 11 MbCFEM proteins were identified from *Marssonina brunnea* f. sp. *multigermtubi* (MULT), a hemibiotrophic pathogenic fungus on poplars that causes severe leaf diseases. Phylogenic analysis showed that the 11 proteins (MbCFEM1-11) were divided into three clades based on the trans-membrane domain and the CFEM domain. Sequence alignment and WebLogo analysis of CFEM domains verified the amino acids conservatism therein. All of them possess eight cysteines except MbCFEM4 and MbCFEM11, which lack two cysteines each. Six MbCFEM proteins with a signal peptide and without trans-membrane domain were considered as candidate effectors for further functional analysis. Three-dimensional (3D) models of their CFEM domains presented a helical-basket structure homologous to the crucial virulence factor Csa2 of *Candida albicans*. Afterward, four (MbCFEM1, 6, 8, and 9) out of six candidate effectors were successfully cloned and a yeast signal sequence trap (YSST) assay confirmed their secretion activity. Pathogen challenge assays demonstrated that the transient expression of four candidate MbCFEM effectors in *Nicotiana benthamiana* promoted *Fusarium proliferatum* infection, respectively. In an *N. benthamiana* heterogeneous expression system, MbCFEM1, MbCFEM6, and MbCFEM9 appeared to suppress both BAX/INF1-triggered PCD, whereas MbCFEM8 could only defeat BAX-triggered PCD. Additionally, subcellular localization analysis indicated that the four candidate MbCFEM effectors accumulate in the cell membrane, nucleus, chloroplast, and cytosolic bodies. These results demonstrate that MbCFEM1, MbCFEM6, MbCFEM8, and MbCFEM9 are effectors of *M. brunnea* and provide valuable targets for further dissection of the molecular mechanisms underlying the poplar-*M. brunnea* interaction.

## Introduction

*Marssonina brunnea* (Ellis & Everh.) Magnus is one of the most pathogenic fungi in poplar-growing areas all over the world (Spiers, 1984; Call and St. Clair, 2017; Zhang et al., 2018a; Xiong et al., 2021). It usually causes severe *Marssonina* leaf disease of poplars (MLDP) such as leaf spots and early defoliation (Beare et al., 1999; Erickson et al., 2004; Zhang et al., 2018b). As a result, the average annual wood production loss can reach 30% (Wu, 2012). According to the modes of conidium germination, *M. brunnea* can be subdivided into two host-adapted formae speciales namely *M. brunnea* f. sp. *monogermtubi* (MONO) and *M. brunnea* f. sp. *multigermtubi* (MULT) (Han et al., 1998; Han et al., 2000). The conidium of the MONO germinates only one germ tube, whereas the conidium of the MULT germinates two to three germ tubes (Li and Han, 2006). Although the two speciales show high similarity in the infection process as hemibiotrophic fungal pathogens, hosts of the two speciales have no overlap in nature (Spiers, 1989). In nature, the MONO infects *Populus* sect. *Aigeiros* (Aig) while MULT infects *Populus* sect. *Leuce Duby* (Leu). Due to the global economic significance of the Leu poplars, efforts have been made to investigate the pathogenesis of *M. brunnea*, and certain effectors of the MULT have been dug up (Cheng et al., 2010; Jiang et al., 2014). Despite this, the knowledge about the effectors of *M. brunnea* is still insufficient.

Plant immunity has become a critical topic in the study of the interaction between plants and pathogens (Jones and Dangl, 2006; Cesari, 2018; Han, 2019). Plants traditionally developed two relatively independent layers of immunity, pathogen or microbe-associated molecular patterns (PAMPs/MAMPs) triggered immunity (PTI) and effector-triggered immunity (ETI) (Tsuda and Katagiri, 2010; Wang et al., 2019; Yuan et al., 2021). More recently, growing evidence has revealed that PTI and ETI work synergistically with each other, and share largely overlapping signaling networks and downstream response (Ngou et al., 2020; Yuan et al., 2020). Plant pathogens could secrete effector proteins into the apoplast and cytoplasm of plants during infection to interfere with signaling pathways in which they act to suppress plant immunity (He et al., 2020; Ceulemans et al., 2021). As an integral aspect of plant-pathogen interaction, many families of pathogen-secreted effectors have been successfully screened and identified (Dou and Zhou, 2012; Yuan et al., 2020; Chang et al., 2022). Some fungal effector proteins possess conserved motifs such as the RXLR (with conserved N-terminal arginine, random amino acid (aa), leucine, and arginine) motif, the CRN motif, and CFEM domain (Fan et al., 2018; Liu et al., 2019). A major class of cytoplasmic effectors containing a secretion signal and a following N-terminal RxLR domain exists in oomycetes. Numerous RXLR effectors in *Phytophthora sojae* have been verified the function of suppressing programmed cell death (PCD) in *Nicotiana benthamiana* triggered by the mouse protein BAX and the *Phytophthora infestans* elicitin infestin 1 (INF1) (Wang et al., 2011; Deb et al., 2018). Some RXLR effectors induce plant defense responses on the contrary but still contribute to virulence (Chen et al., 2019; Situ et al., 2020). CRNs are also a large family of effector proteins, which trigger crinkling phenotype on leaves in ectopic expression (Schornack et al., 2010; Amaro et al., 2017). Many CRN effectors have been identified and proved to promote virulence in both oomycetes and fungi (van Damme et al., 2012; Ai et al., 2021). Studies about the underlying mechanism behind different kinds of effectors have also been carried out. For example, PexRD12/31 family of RXLR-WY effectors in *P. infestans* were found associate with components of the vesicle trafficking machinery (Petre et al., 2021). *P. infestans* splicing regulatory effectors (SREs) are involved in the plant AS machinery and subsequently modulates plant immunity (Huang et al., 2020). The RXLR effector Avh238 from *P. sojae* can suppress ethylene biosynthesis by disrupting the Type2 GmACSs in soybean and promote infection (Yang et al., 2019). Avh94 from *P. sojae* can manipulates host jasmonic acidsignaling to accelerate infection (Zhao et al., 2022). CRN108 from *P. sojae* can enhance host susceptibility by restraining the expression of plant heat shock proteins (Song et al., 2015). AeCRN13 of *Aphanomyces euteiches* and BdCRN13 of *Batrachochytrium dendrobatidis* can induce DNA damage in the nucleus to facilitate infection (Ramirez Garcés et al., 2015).

CFEM effectors are also a broad class of fungal-specific conserved effector proteins that symbolically containing Common in Fungal Extracellular Membrane (CFEM) domain (Kulkarni et al., 2003; Zhang et al., 2015). In-depth studies on model fungi species such as *Colletotrichum graminicola* and *Magnaporthe oryzae* have been conducted (Dezwaan et al., 1999; Jing et al., 2016). Five CFEM effectors in *C. graminicola* were confirmed capability to suppress BAX-triggered PCD in *N. benthamiana*. A total of 19 proteins in *M. oryzae* were identified and Pth11 in particular was proved as an important factor during infection (Kou et al., 2016; Gong et al., 2020). As a prominent family of effectors, the number of CFEM has a positive correlation with fungi pathogenicity since pathogenic species contain more CFEM proteins than nonpathogenic ones (Gong et al., 2020). CFEM domain is usually about 60 amino acids long and possesses 8 cysteine residues at distinctive intervals as the PxC[A/G]x_2_Cx_8-12_Cx_1-3_[x/T] Dx_2-5_CxCx_9-14_Cx_3-4_Cx_15-16_ (x as any residue with its range shown) sequence (Choi and Dean, 1997). CFEMs are mainly glycosylphosphatidylinositol (GPI)-anchored proteins that are attached to the fungal cell wall. Three CFEM effectors possessing the C-terminal GPI anchor can affect the stability of the cell wall (Vaknin et al., 2014). A large amount of CFEM-GPCRs (G-protein coupled receptors) has been unearthed in *M. oryzae.* Among them, WISH and Pth11 have been demonstrated to contribute to *M. oryzae* infection (Nazmiara and Subhankar, 2017; Weis and Kobilka, 2018). BcCFEM1 from *Botrytis cinerea*, which contains a GPI-anchored site, is associated with virulence and viability including conidial production and stress tolerance (Zhu et al., 2017). According to a review on the evolution of over 100 fungal species, CFEM shows unique to fungi and tends to be more common among pathogenic fungi (Zhang et al., 2015). While CFEM effectors play a significant role in pathogenicity for fungi, no CFEM effector has been identified in *M. brunnea* that the lack of identification and functional analysis are still obvious.

In this study, we predicted 11 CFEM proteins from the *M. brunnea* genome by bioinformatic analysis. Based on the definition of effector proteins, six of these were considered as candidate effectors. Then, four of six candidate effectors were successfully amplified, and conducted a functional analysis using the *Agrobacterium*-mediated transient expression system in *N. benthamiana.* To the best of our knowledge, this is the first time for CFEM effectors to be identified with an exploration of the function in *M. brunnea*, which lays the groundwork for further research on the mechanism of interaction between *M. brunnea* and its host poplars.

## Materials and methods

### Plants, microbes, and growth conditions

*N. benthamiana* was grown in a climatic chamber at 23°C (light) and 21°C (dark) with a 16 h light period. The branches of *Populus euramericana* cv. I-214 were grown in pots at 22°C with a 12 h photoperiod. The wild-type *M. brunnea* f. sp. *multigermtubi* strain, J4, was cultured on potato sucrose medium (PSA: 20% potato, 2% sucrose, and 1.5% agar) at 25°C in dark. The *Escherichia coli* strain DH5α and *Agrobacterium tumefaciens* strain GV3101 were cultured on Luria-Bertani solid medium (LB: 5% yeast extract, 10% tryptone, 10% NaCl, and 1.5% agar) and shaking-cultured at 200rpm in liquid LB at 37°C and 28°C, respectively. Yeast YTK12 strain was grown on yeast extract peptone dextrose medium (YPD: 1% yeast extract, 2% peptone, 2% glucose, 0.003% adenine sulphate, and 1.5% agar) at 30°C in dark.

### Bioinformatic identification of CFEM proteins in *M. brunnea*

To identify CFEM proteins in *M. brunnea* f. sp. *multigermtubi*, the CFEM effector protein ACI1 from *M. oryzae* was used as the query. The genome database AFXC00000000.1 of *M. brunnea* f. sp. *multigermtubi* MB_m1 was searched using Basic Local Alignment Search Tool algorithms (BLASTP) with a threshold of E-value<1e^–10^. All the obtained proteins were scrutinized for the existence of CFEM domain by the Pfam on the SMART website (http://smart.embl-heidelberg.de/) and only sequences with CFEM domains were presented. Signal peptides of these candidate proteins were predicted on SignalP Server (4.0, 5.0, and 6.0, respectively) (http://www.cbs.dtu.dk/services/SignalP/). Transmembrane regions of the MbCFEM proteins were predicted by TMHMM (http://www.cbs.dtu.dk/services/TMHMM/). Subcellular localization of the MbCFEM proteins was predicted using TargetP 2.0 Server (https://services.healthtech.dtu.dk/service.php?TargetP-2.0) and Wolf Psort (https://wolfpsort.hgc.jp/). The GPI anchor was predicted by PredGPI predictor (http://gpcr2.biocomp.unibo.it/gpipe/pred.htm).

### Multiple sequence alignments and phylogenetic analysis

The phylogenetic tree of MbCFEM proteins was constructed by the neighbor-joining method using MEGAX. To examine the conserved amino acids, a multiple protein sequence alignment upon the CFEM domains of all the MbCFEM proteins was performed using ClustalW (Han et al., 2016). Csa2 (XP_715426.1) from *Candida albicans* and ACI1 (AY166602.1) from *M. oryzae* were added as templates. The alignment results were modified and presented using DNAMAN and WebLogo (http://weblogo.berkeley.edu/logo.cgi). To show the domain architecture in each protein, all the domains, including signal peptides, CFEM domains, and trans-membrane regions that were predicted in bioinformatic analysis, were highlighted.

### Protein model analysis of candidate MbCFEM effectors with Phyre2 Server

A total of six MbCFEM proteins were selected as candidate effectors due to the presence of signal peptide at the N terminal and lack of trans-membrane region. For further exploring the structure of their CFEM domains, Phyre2 (http://www.sbg.bio.ic.ac.uk/phyre2) was employed to construct the predicted CFEM structures of candidate MbCFEM effectors and built the protein models. The sequences of the CFEM domain were blasted with PSI-Blast to find homologies and the Hidden Markov Model (HMM) structures were built. To conduct the functional analysis, all the candidate effectors were then amplified from the cDNA of *M. brunnea* f. sp. *multigermtubi* strain J4 by PCR with the corresponding primer pairs in **Supplementary Table S1**.

### Expression pattern analysis of candidate *MbCFEM*s during infection with qRT-PCR

To examine the expression of candidate *MbCFEM*s during infection, a qRT-PCR assay was conducted to analyze transcript levels at different infection stage (Cheng et al., 2014). For inoculation, a large amount of fresh leaves were cut off from two-week old cutting seedlings of the susceptible poplar clone I-214 and placed onto sterilized 1% water agar with abaxial surface up. Conidia of *M. brunnea* f. sp. *multigermtubi* strain, J4, were harvested from the ten-day culture, suspended in deionized water, and adjusted to 10000 spores/mL. For each leaf, 200 μL of conidia were evenly sprayed to the up-side of leaves. The Total RNA of 0-8 days post inoculation (dpi) samples were extracted and for each sample, reverse transcription was conducted using 2 mg of total RNA. The qRT-PCR was performed using specific primers in **Supplementary Table S1**. *Elongation factor 1-a* was used as an internal control gene and to normalize values obtained in qRT-PCR.

### Functional validation of predicted signal peptides of candidate MbCFEM effectors

To verify the secretion ability of the candidate proteins, a yeast signal sequence trap (YSST) assay was performed. The predicted signal peptide sequences of the candidate proteins were amplified by PCR with the corresponding primer pairs in **Supplementary Table S1** and subsequently introduced into pSUC2 using EcoRI and XhoI restriction sites (Jacobs et al., 1997; Yin et al., 2018). All the transformants were then screened on selective media CMD-W, and positive clones were transferred to YPRAA plates. As a yeast vector carrying an invertase gene (*SUC2*) that lacks initiator methionine codon (Met) and signal peptide (SP), transformed pSUC2 with native Met and SP can support SUC2-yeast YTK12 to grow on raffinose after 3 days incubation. Avr1b was used as a positive control and empty pSUC2 as a negative control. The color reaction of the reduction of 2,3,5-Triphenyltetrazolium Chloride (TTC) to insoluble red-colored 1,3,5-Triphenylformazan (TPF), which is catalyzed by invertase enzymatic activity, was used to detect the invertase activity of the transformants. Transformants were cultured in CMD-W liquid medium and shaken at 220 rpm for 24 h at 30°C. About 1.5 mL of cell suspension was concentrated and re-suspended to a volume of 400 μL after being washed with sterile distilled water three times. The suspension was then added into 2 mL of 5% sucrose solution (w/v). All the mixture was finally moved to a glass test tube and added 0.267 mL 0.1% TTC to react at 220 rpm for 30 min at 37°C. Colorimetric changes were observed and indicated the secretion ability of the predicted signal peptides.

### Promoting Fusarium proliferatum infection on *N. benthamiana*

As a highly effective expression vector, Potato Virus X (PVX) vector pGR107 with a 3×flag-tag was employed to analyze the virulence of the candidate MbCFEM effectors (Qiao et al., 2013). The open reading frame (ORF) sequences of candidate MbCFEM effectors without the signal peptide (-SP) and with an additional engineered ATG were amplified with the corresponding primer pairs in **Supplementary Table S1**, cloned into PGR107, and transformed into GV3101. To examine whether the MbCFEM candidate effectors can accelerate infection, a significantly large number of *N. benthamiana* leaves were then challenged with *F. proliferatum*, which is recently identified as a fungal pathogen of tobacco (Li et al., 2017). After multiple pathogenicity tests on tobacco, *F. proliferatum* can develop infection and cause leaf spots stably, obviously, and quickly, thereby fully meeting the needs of this pathogen challage assay and utilizing it. The pGR107-MbCFEM-SPs were overexpressed on the right half of the leaves while the empty vector on the left half was a negative control. The *Agrobacterium* suspension was adjusted to OD_600 =_ 0.4. For inoculation on *N. benthamiana*, 5-mm disks of 5-day growth mycelium were inoculated on the leaf back 24 h after infiltration. For each candidate MbCFEM effectors, 10 leaves were utilized, and the experiments were repeated three times. Leaves were photographed under UV light at 3 dpi and then stained with trypan blue to display the lesion area. The lesion area was measured using ImageJ. Western blot was performed to confirm the expression of all the candidate MbCFEM effectors using an anti-HA antibody.

### Suppression of PCD triggered by BAX/INF1

pGR107-MbCFEM-SPs were also employed to test whether the four candidate effectors can suppress programmed cell death triggered by BAX or INF1 (Xiang et al., 2016). For the *Agrobacterium*-mediated transient expression, GV3101 carrying pGR107-MbCFEM-SPs were infiltrated into tobacco leaves and then, 24 h later, the same infiltration site was challenged with GV3101 carrying BAX. Empty pGR107 with BAX or INF1 was used as a negative control. All the *Agrobacterium* suspensions were adjusted to OD_600 =_ 0.4. For each candidate MbCFEM effectors, 10 leaves were utilized, and the experiments were repeated three times. The degree of PCD was observed at 4 dpi. Western blot was performed to confirm the expression of all the candidate MbCFEM effectors using an anti-HA antibody.

### Subcellular localization of candidate MbCFEM effectors

Subcellular localization was conducted by agroinfiltration method using a green fluorescent protein (GFP) expression vector pBinGFP4. The open reading frame (ORF) sequences of candidate MbCFEM effectors without the signal peptide (-SP) and with an additional engineered ATG were cloned into pBinGFP4 and transformed into GV3101 (Jin et al., 2019). N-terminal GFP-tagged pBinGFP4-MbCFEM-SPs were then transiently expressed in *N. benthamiana* leaves. All the *Agrobacterium* suspensions were adjusted to OD_600 =_ 0.25. For each candidate MbCFEM effectors, 10 leaves were utilized, and the experiments were repeated three times. Fluorescence signals were detected by a Zeiss confocal microscopy after 2 dpi. The nucleus was marked by 4’,6-diamidino-2-phenylindole (DAPI), which was infiltrated into the leaves 15 min before fluorescence signal detection, and captured with an excitation wavelength of 350 nm and emission of 461 nm. The chloroplast was presented by its autofluorescence and captured with an excitation wavelength of 470 nm and emission of 680 nm. GFP was captured with an excitation wavelength of 488 nm and an emission of 510 nm. Western blot was performed to confirm the expression of all the candidate MbCFEM effectors using an anti-GFP antibody

## Results

### Bioinformatic identification and analysis of CFEM-containing proteins in *M. brunnea*

A total of 11 CFEM proteins (MbCFEM1-11) were identified in the *M. brunnea* J4 genome using BLASTP analysis. The length of these proteins ranged from 167 aa (MbCFEM8) to 919 aa (MbCFEM10). The CFEM domains were further confirmed by SMART analysis. Except for MbCFEM10, all the CFEM domains were located near the N-terminus in protein. The signal peptides were subsequently predicted on SignalP Server and all the MbCFEMs have a predicted signal peptide on the N-terminus. MbCFEM4 lacks a probable signal peptide on both the 4.0 and 5.0 server and the predicted signal peptide of MbCFEM6 is partially overlapped with its CFEM domain both on the 5.0 and 6.0 server. To minimize the discrepancy, predictions of the 6.0 server were presented for all the other MbCFEMs while predictions of the 4.0 server for MbCFEM6. The protein ID number and all the analyses are shown in **Table 1**.

**Table 1:** The identification of Common in Fungal Extracellular Membrane (CFEM) protein in Marssonina brunnea.

Name	Protein ID	Amino acids (aa)	Position of CFEM domain (aa)	SP cleavage^1^)	Cys no.	Cys% in matured protein	mTP^2^)	SP	Other^3^)	TM no.^4^)	Loc	GPI-Anchored^7^)
MbCFEM1	XP_007293007.1	235	23-89	17-18	8	0.034	0	0.9997	0.0002	0	S^5^)	T^211^/V^235^
MbCFEM2	XP_007294420.1	173	24-90	18-19	8	0.046	0	0.9998	0.0002	0	S	T^150^/L^173^
MbCFEM3	XP_007293491.1	392	30-99	25-26	9	0.023	0.0006	0.9996	0.0004	1	S	–
MbCFEM4	XP_007289105.1	455	55-121	51-52	13	0.029	0.0001	0.71	0.29	7	-^6^)	–
MbCFEM5	XP_007289520.1	544	30-94	26-27	18	0.033	0.0008	0.9998	0.0002	7	S	–
MbCFEM6	XP_007292888.1	271	18-85	17-18^8^)	8	0.03	0	0.9996	0.0004	0	S	S^247^/L^271^
MbCFEM7	XP_007295384.1	453	26-90	18-19	16	0.033	0.0007	0.9998	0.0002	7	S	–
MbCFEM8	XP_007292405.1	167	20-85	17-18	8	0.048	0	0.9998	0.0002	0	S	G^143^/L^167^
MbCFEM9	XP_007294196.1	239	36-99	18-19	9	0.033	0	0.9997	0.0003	0	S	G^215^/L^239^
MbCFEM10	XP_007291058.1	919	565-631	20-21	21	0.021	0	0.9997	0.0003	0	S	N^896^/L^919^
MbCFEM11	XP_007288866.1	653	27-91	22-23	30	0.044	0	0.9997	0.0003	7	S	–

^1)^SP cleavage, cleavage site of signal peptide (SP) in the MbCFEM proteins.

^2)^mTP, a mitochondrial targeting peptide prediction.

^3)^Other, any other localization.

^4)^TM, transmembrane domain.

^5)^S, secretory pathway.

^6)^–, no prediction.

^7)^GPI-anchored, a GPI-modification site prediction.

^8)^All the SP cleavage sites presented were predicted by SignalP Server 6.0 except that of MbCFEM6 by SignalP Server 4.0.

**Figure 1:**
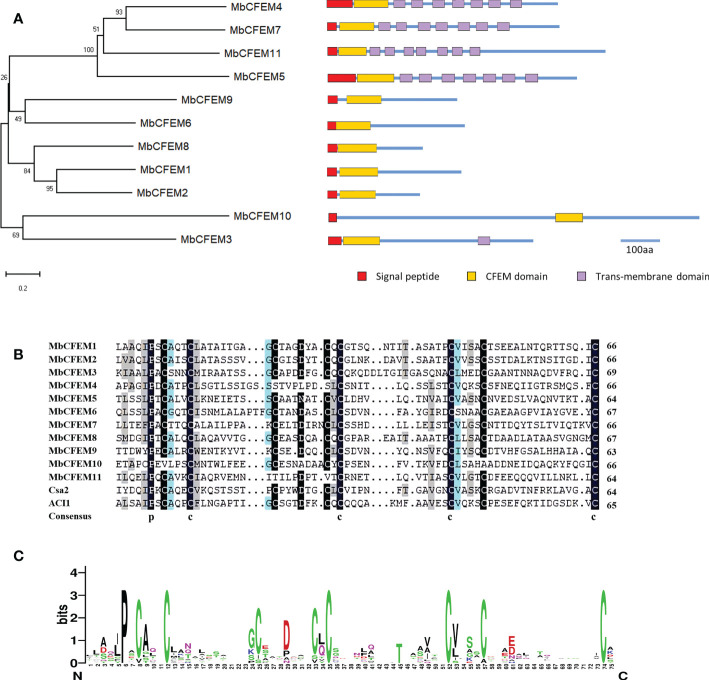
Bioinformatics analysis of Common in Fungal Extracellular Membrane (CFEM) proteins in *M. brunnea.* **(A)** Phylogenetic analysis of Common in Fungal Extracellular Membrane (CFEM) proteins in *M. brunnea*. A neighbor-joining phylogenetic tree was constructed based on the amino acid sequences of MbCFEM proteins. The numbers at nodes represent the percentage of their occurrence in 1000 bootstrap replicates; the nodes supporting less than 50% are not shown. Maps of the corresponding MbCFEM proteins are shown on the right. The red box represents the signal peptide, the yellow box represents the CFEM domain, and the purple box represents the trans-membrane domain. Scale bar = 100 amino acids (aa). **(B)** Multiple amino acid sequence alignment in Fungal Extracellular Membrane (CFEM) domains from 11 MbCFEM proteins. Alignments were performed with ClustalW and modified with DNAMAN. Conserved amino acids are highlighted in black, blue, and grey in descending order of conservatism. **(C)** WebLogo of Common in Fungal Extracellular Membrane (CFEM) domains from 11 MbCFEM proteins. The conservatism degree of amino acids was indicated by the size through WebLogo.

A neighbor-joining phylogenetic tree was generated based on the sequences of MbCFEM proteins. Four MbCFEMs (MbCFEM4, 5, 7, and 11), each of which possesses seven trans-membrane regions, were grouped into one clade; Five MbCFEMs (MbCFEM1, 2, 6, and 8) with no trans-membrane region contained were assembled into one clade; MbCFEM3 with one trans-membrane region and MbCFEM10 with no trans-membrane region were additionally classified into one clade (**Figure 1A**).

For further analysis of the CFEM domains, a multiple sequence alignment was constructed by ClustalW to search for the conserved amino acids (**Figure 1B**). Two well-known CFEM proteins, Csa2 from *C. albicans* and ACI1 from *M. oryzae* were selected to be references. Except for MbCFEM4, MbCFEM10, and MbCFEM11, which both lack two conserved cysteines, most CFEM domains contain eight characteristically spaced cysteines that can form four disulfide bonds to help stabilize the structure of the domain. MbCFEM4 and MbCFEM11 both lack the third and fourth cysteine, and MbCFEM10 lacks the first and seventh cysteine. There are also several relatively conserved residues among them such as proline (position 6), alanine (9), glycine (24), aspartic acid (29), and valine (53). WebLogo analysis also confirmed the results (**Figure 1C**). The composition of amino acids may influence the structures of CFEM domains and contribute to functions that interpose the plant-pathogen interaction.

### Detection of helical-basket shape in the model structure of CFEM domain

**Figure 2:**
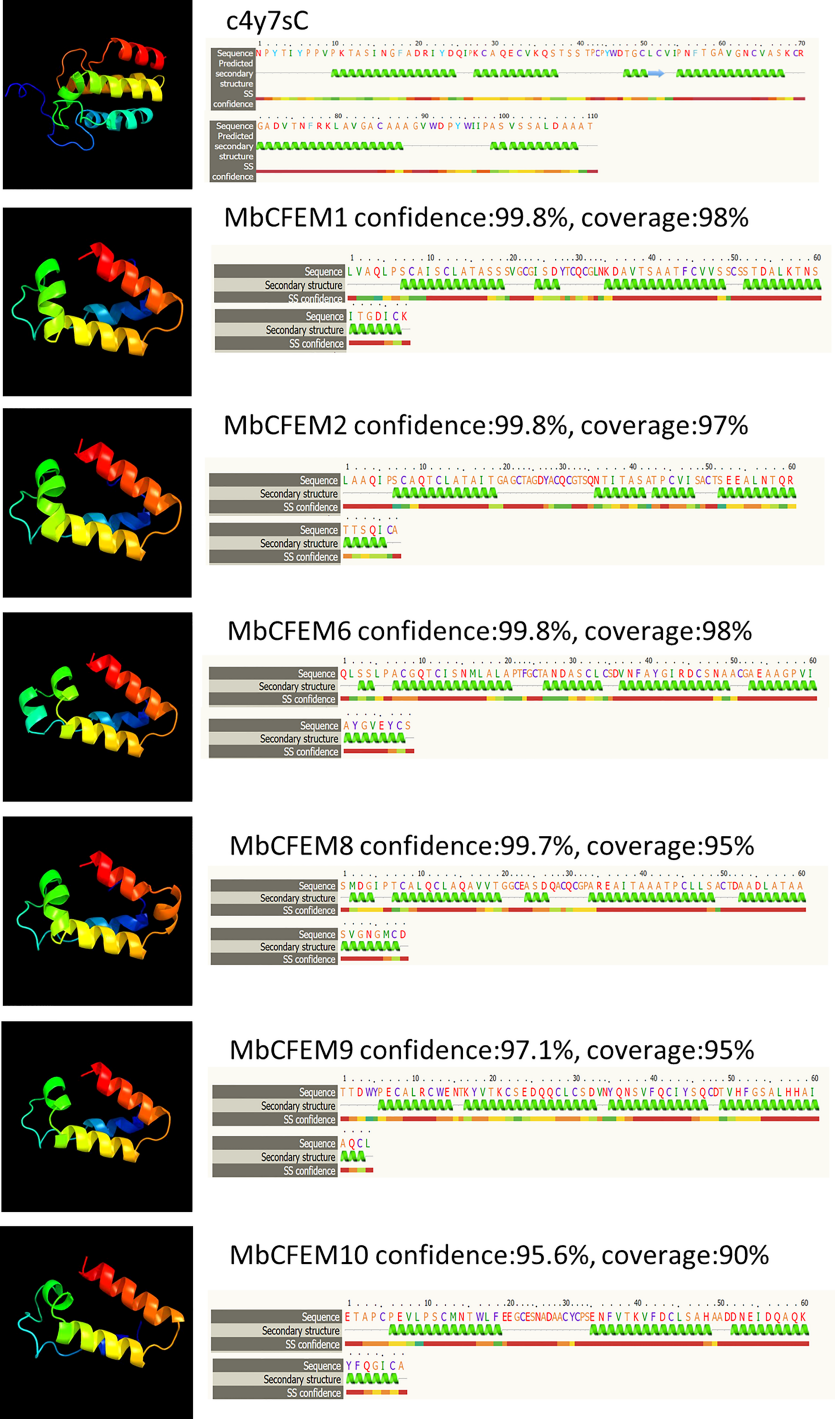
Analysis of structural models of candidate MbCFEM effectors by the Phyre2 Server. The amino acid residues of these effectors were modeled on the crystal structure of the Common in Fungal Extracellular Membrane (CFEM) domain c4y7sC from *C. albicans* Csa2 with more than 95% confidence. The amino acid sequences of candidate MbCFEM effectors are shown in the top line. The sequences on the next line show residues, which were colored according to a property-based scheme: yellow (A, S, T, G, P: small/polar), green (M, I, L, V: hydrophobic), red (K, R, E, N, D, H, Q: charged) and purple (W, Y, F, C: aromatic+cysteine). Green helices represent α-helixes, blue arrows represent β-strands and faint lines represent coils.

A total of six MbCFEM proteins (MbCFEM1, 2, 6, 8, 9, and 10) were finally considered as candidate effectors according to the definition of pathogen effectors (with a signal peptide and no trans-membrane region). Three-dimensional (3D) model structures of the CFEM domains from all six candidate effectors were constructed and conducted homology analysis by Phyre2. All the CFEM domains showed a high similarity to Csa2 (c4y7sC in *C. albicans*), which is the only CFEM protein possessing a 3D crystal structure, and were modeled with the confidence of more than 95% and coverage of more than 90%. The CFEM domain of Csa2 possesses a helical-basket shape formed by its 6 α-helices and 1 β-strand, which contains an elongated N-terminal loop as a handle. CFEM domains of the six candidate effectors also have helical basket-like structures similar to Csa2 (**Figure 2**). MbCFEM6 and MbCFEM8 contain 5 α-helices, MbCFEM1 and MbCFEM9 contain 4 α-helices, and MbCFEM2 and MbCFEM10 contain 3 α-helices.

### Cloning six candidate MbCFEM effectors from the *M. brunnea* f. sp. *multigermtubi* strain J4

A total of four candidate MbCFEM effectors (MbCFEM1, 6, 8, and 9) were successfully cloned from the *M. brunnea* f. sp. *multigermtubi* strain J4 cDNA, and their nucleotide sequences were submitted to NCBI with accession numbers (GenBank nos. OP410744, OP410745, OP410746, and OP410747, respectively). We failed to amplify MbCFEM2 and MbCFEM10, despite numerous trials. Nucleotide sequence analysis verified that MbCFEM6 and MbCFEM8 had the same length and sequence compared with the published genome sequence. However, MbCFEM1 has two 1 nt mutations, one 12 nt insertion, and one 105 nt deletion; and MbCFEM9 has one 48 nt deletion (**Supplementary Table S2**), both of which resulted in shorter ORFs. Although, the signal peptides and CFEM domains were not under the influence and no trans-membrane region newly emerged in cloned MbCFEM1 and MbCFEM9. The results were shown in **Supplementary Table S2**.

### Candidate *MbCFEMs* are highly expressed in the biotrophic stage of the *M. brunnea* f. sp. *multigermtubi* strain J4

**Figure 3:**
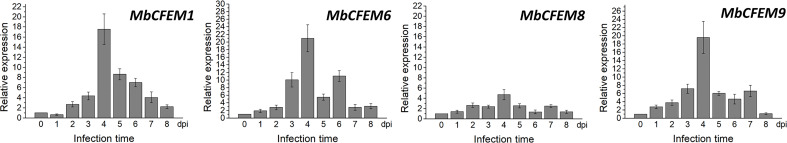
Transcript profiles of four candidate *MbCFEMs* in *M. brunnea*-infected poplar leaves at 0, 1, 2, 3, 4, 5, 6, 7, and 8 days post inoculation (dpi). Relative expression levels of four candidate MbCFEMs (MbCFEM1, MbCFEM6, MbCFEM8, and MbCFEM9) were determined by quantitative reverse-transcriptase polymerase chain reaction (qRT-PCR) using the *M. brunnea* housekeeping gene *EF-1α* (*elongation factor 1-α*) as an internal standard. Values are means ± standard deviations (as error bars) (*n* = 3). This experiment was repeated three times with similar results.

During the infection of *M. brunnea* f. sp. *multigermtubi* strain J4 on *Populus euramericana* cv. I-214, all the four candidate *MbCFEM*s showed high expression in the infection stage (**Figure 3**). The transcript levels of *MbCFEM1*, *MbCFEM6* and *MbCFEM9* presented a uniform expression pattern. These three *MbCFEM*s maintained low expression levels from 0 to 2 dpi and were induced at 3 dpi. The expression peak was then precipitously reached at 4 dpi while the expression levels suddenly declined at 5 dpi. In particular, the expression levels of *MbCFEM6* showed an obvious rebound at 6 dpi on its way returning to the low expression levels from 6 to 8 dpi. *MbCFEM9* also presented an upturn at 7 dpi although it is slight. In contrast to these three *MbCFEMs*, *MbCFEM8* was induced in the early days but showed lower transcript levels during the whole infection, though it also reached an expression peak at 4 dpi and rose again at 7 dpi. This result indicates that transcription of *MbCFEM1*, *MbCFEM6*, *MbCFEM8* and *MbCFEM9* are all induced during the infection of *M. brunnea* f. sp. *multigermtubi* strain J4 on *Populus euramericana* cv. I-214.

### Signal peptides of four candidate MbCFEM effectors possess secretion activation

**Figure 4:**
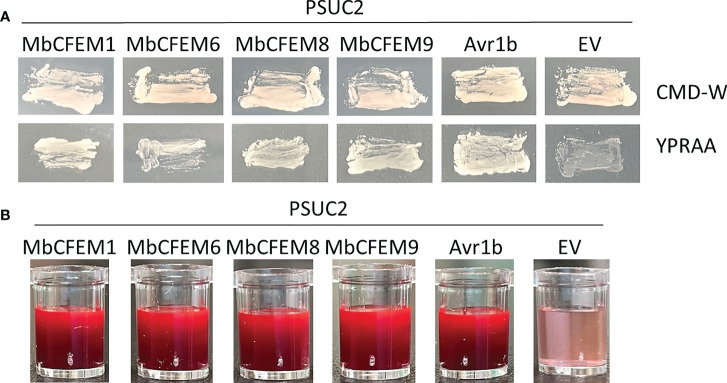
Functional evaluation of the signal peptides from four candidate MbCFEM effectors. **(A)** The secretion function of the predicted signal peptides was proved by employing a yeast secretion system with *Saccharomyces cerevisiae* strain YTK12. PSUC2 carrying signal peptides from four MbCFEM candidate effectors and the positive control Avr1b could all make YTK12 survive on CMD-W and YPRAA medium, while YTK12 containing empty PSUC2 as a negative control failed to grow on YPRAA. **(B)** The invertase activity was verified by the reduction of 2,3,5-triphenyltetrazolium chloride (TTC) to the insoluble, red-colored 1,3,5-triphenylformazan (TPF). PSUC2 carrying signal peptides from candidate MbCFEM effectors and the positive control Avr1b successfully turned the reaction mixture to bright and thick red while the negative control stayed pink as the original.

The signal peptides (SP) of the four obtained candidate MbCFEM effectors (MbCFEM1, 6, 8, and 9) were then successfully amplified, introduced into pSUC2, and transformed into a secreted invertase lacking yeast strain YTK12. The transformants with MbCFEM SP presented clear streaks on both CMD-W plates and YPRAA plates like the positive control Avr1b, while the negative control showed light and weak growth (**Figure 4A**). During the further detection of the enzyme activity, the MbCFEM SP transformants successfully reduced the 2,3,5-Triphenyltetrazolium Chloride (TTC) to apparent insoluble red-colored 1,3,5-Triphenylformazan (TPF) (**Figure 4B**). These results demonstrated that the signal peptides of candidate MbCFEM effectors were functional and confirmed the accuracy of the secretion activation of the four candidate MbCFEM effectors.

### The candidate MbCFEM effectors promote *F. proliferatum* infection on *N. benthamiana*

**Figure 5:**
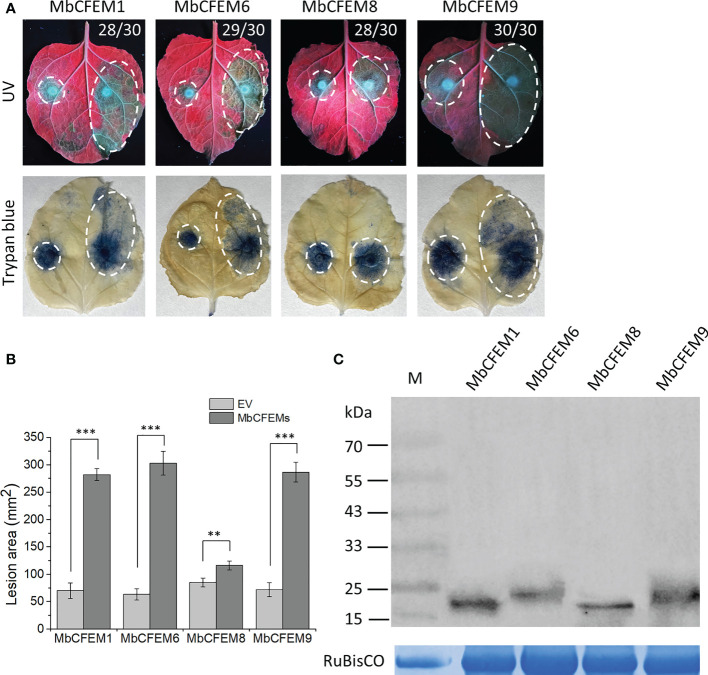
Expression of four candidate MbCFEM effectors in *Nicotiana benthamiana* enhances *Fusarium proliferatum* infection. **(A)** Function of the candidate MbCFEM effectors to accelerate the infection was examined through the infection of *F. proliferatum* on *N. benthamiana*. Tobacco leaves were infiltrated with *Agrobacterium* pGR107 carrying each candidate MbCFEM effector on the right side and an empty vector as a control on the left side. Leaves were subsequently inoculated with *F. proliferatum* mycelium disks (5-mm) 24 h after the agroinfiltration. The results were photographed 3 days post inoculation (dpi) both under UV (photographed from the abaxial side) and after staining with trypan blue (photographed from the adaxial side). Numbers, e.g., 28/30, indicate that 28 of 30 infiltrated leaves exhibited larger lesion on the MbCFEMs-overexpressing side. **(B)** Average areas of the lesions were calculated by ImageJ and all the data are presented as the mean of three biological replicates ± SD (one-way ANOVA, Tukey’s Multiple Comparison Test, ** and *** represent statistical significance at *P* < 0.01 and *P* < 0.001, respectively). **(C)** Western blotting was performed to confirm the expression of all the four candidate MbCFEM effectors using an anti-HA antibody. The protein gel was stained with Coomassie blue as the loading control. Experiments were repeated three times with similar results.

The function of the MbCFEM candidate effectors to accelerate virulence was examined through the infection of *N. benthamiana* by *F. proliferatum*. The grey lesion excited by UV was emitted from pathogen lesions on *N. benthamiana* and could indicate the degree of infection. Leaf areas that overexpressed MbCFEM1, MbCFEM6, and MbCFEM9 had infecting lesions rapidly spread in 3 d while the negative control only presented slight lesions. MbCFEM8 only showed a mild promotion to infection (**Figure 5A**). After being stained with trypan blue and decolorized with 100% ethanol, the lesion areas were displayed more clearly (**Figure 5A**). The areas were further measured using ImageJ and analyzed with a bar chart, resulting that all the candidate MbCFEM effectors (MbCFEM1, 6, 8, and 9) significantly surpassed negative control on the promotion to infection (**Figure 5B**). These results indicate that these candidate MbCFEM effectors can promote *F. proliferatum* infection on *N. benthamiana*.

### The candidate MbCFEM effectors suppress programmed cell death in *N. benthamiana*

**Figure 6:**
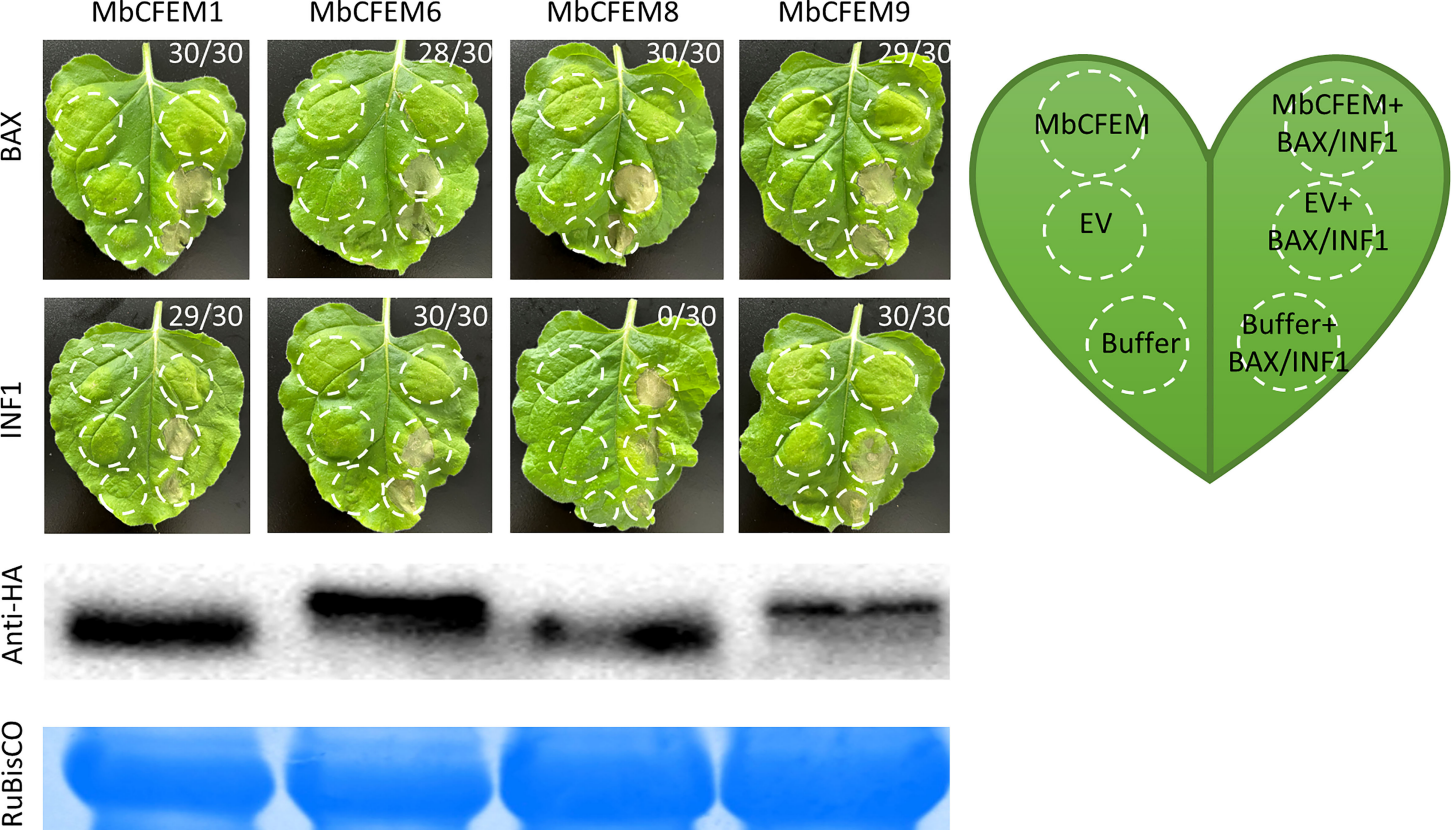
Suppression of BAX/INF1-triggered cell death on *N. benthamiana* leaves. The *Agrobacterium* suspension carrying each of the tested candidate MbCFEM effectors (MbCFEM1, MbCFEM6, MbCFEM8, and MbCFEM9) was infiltrated into tobacco leaves followed by the infiltration of the mouse protein BAX or the *Phytophthora infestans* elicitin infestin 1 (INF1) in the same position after 24 h to examine the suppression function of the hypersensitive response. All the infiltration positions were illustrated in the model leaf. Agroinfiltration was performed on tobacco leaf with *Agrobacterium tumefaciens* carrying MbCFEMs, MbCFEMs with BAX/INF1, EV (empty vector), EV with BAX/INF1, infiltration buffer, and infiltration buffer with BAX/INF1. EV and infiltration buffer were used as negative controls while EV with BAX/INF1 and infiltration buffer with BAX/INF1 as positive controls. The results were photographed 4 days post inoculation (dpi). Numbers, e.g., 30/30, indicate that 30 of 30 infiltrated leaves exhibited suppression of the cell death triggered by BAX or INF1 on the MbCFEM effectors overexpressed site (MbCFEM+BAX/INF1). Western blot was performed to confirm the expression of all the candidate MbCFEM effectors using an anti-HA (hemagglutinin) antibody. Experiments were repeated three times with similar results.

Some fungal effectors weaken plant immunity responses to strengthen the infection by suppressing programmed cell death (PCD) (Dong et al., 2015). Due to the significance of suppression of defense-related HR in plant cells during infection, BAX/INF1-triggered programmed cell death (BT-PCD/IT-PCD) was employed as an imitation of HR to examine the effector activity against plant immunity (Balint Kurti, 2019). MbCFEM1, MbCFEM6, and MbCFEM9 could completely suppress both BT-PCD and IT-PCD while MbCFEM8 only defeated BT-PCD (**Figure 6**). Above all, these results indicate that the four candidate MbCFEM effectors can suppress PCD that is associated with PTI responses so that they may make primary contribution to promote infection.

### Subcellular localization of the candidate MbCFEM effectors

**Figure 7:**
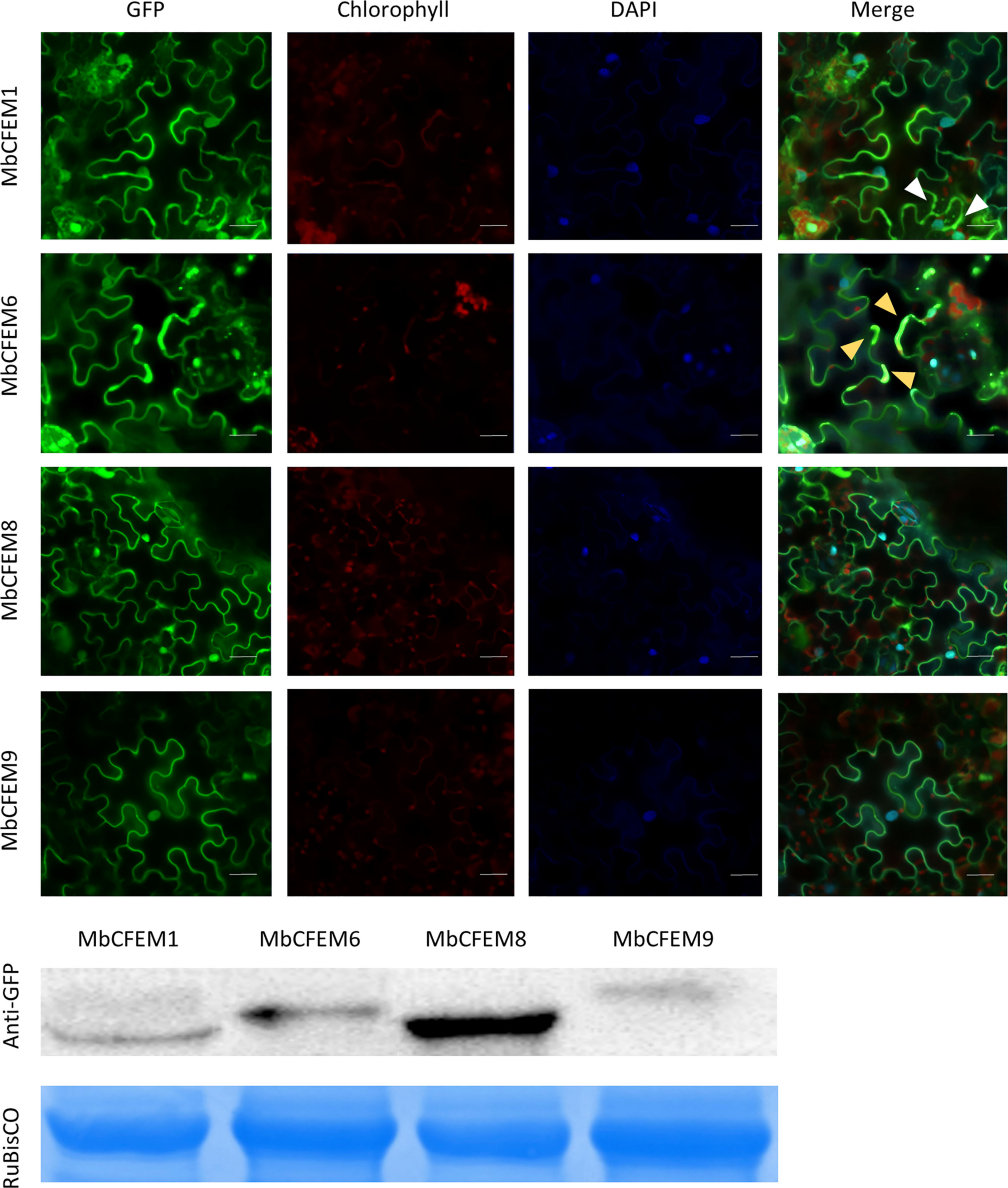
Subcellular localization of four candidate MbCFEM effectors in *N. benthamiana.* N-terminal GFP-tagged MbCFEM effectors were transiently expressed in *N. benthamiana*. 4’,6-diamidino-2-phenylindole (DAPI) was used as the marker of the nucleus. 10 leaves of *N. benthamiana* were used for each candidate MbCFEM effectors and the experiment was repeated three times. The subcellular localization was detected by a confocal laser-scanning microscope and the representative images were present. The DAPI signal, the chlorophyll signal and the GFP signal were captured with an excitation wavelength of 350 nm, 470 nm and 488 nm, respectively, and an emission of 461 nm, 680 nm and 510 nm, respectively. White arrowheads indicate the speckle-like cytosolic bodies (MbCFEM1). Yellow arrowheads indicate the chloroplast (MbCFEM6). Images displayed are overlays of the GFP signal, the chlorophyll signal, the DAPI signal, and the merged field. Scale Bars = 100 μm.

To increase the susceptibility of the host, fungal effectors usually target different organelles and interfere host defense signaling pathway to break the plant immune system (Liu et al., 2014). Therefore, the subcellular localization of effectors plays an important role in the infection mechanism. The four N-terminal GFP-tagged MbCFEM effectors were transiently expressed in *N. benthamiana* to make the subcellular localization clear. All the fluorescence signals were successfully captured in four MbCFEM effectors-expressing *N. benthamiana* leave cells by a confocal laser-scanning microscope (**Figure 7**). The results showed that the GFP signal of all four MbCFEM effectors could be detected in both the nucleus and the cell membrane. In addition to clear nuclear and membrane localization, MbCFEM1 was found to accumulate in speckle-like cytosolic bodies, and MbCFEM6 was partially associated with the chloroplast (**Figure 7**).

## Discussion

There has been a mountain of evidence proving that pathogen effectors are a set of indispensable factors during the interaction between plants and pathogens (Ishikawa et al., 2014; Piasecka et al., 2015; He et al., 2020). Studies on fungal effectors have also been increasing in recent years (Brown et al., 2012; Wang et al., 2022b). As a conserved family of fungal protein groups, CFEM proteins uniquely existed in fungi and proved to be functional effectors that mediate PTI and ETI to assist pathogen survival during the plant-pathogen reaction (Dezwaan et al., 1999; Lamarre et al., 2000; Weissman and Kornitzer, 2004; Guyon et al., 2014; Zhang et al., 2015). In this study, we identified 11 CFEM proteins (MbCFEM1-11), each of which possesses a CFEM domain, in *M. brunnea* (**Table 1**). Most of their CFEM domains locate close to the N-terminus while the one in MbCFEM10 is near the C-terminus (**Figure 1A**), which is a similar condition to *C. graminicola* (Jing et al., 2016). It points out that there may be multiple functions among all the MbCFEMs.

CFEM domain is relatively conserved that it usually contains a typical eight characteristically spaced cysteines to form four disulfide bonds for structure stable (Cai et al., 2022).Many effectors predicted from filamentous pathogens contain small cysteine-rich (SCR) proteins and proved to be functional during infection (Saunders et al., 2012). For example, disulfide-bonded cysteine pair of the *Pyrenophora tritici-repentis* effector ToxA can be internalized into mesophyll cells in sensitive wheat lines (Manning and Ciuffetti, 2005; Sarma et al., 2005). The protein with four disulfide-bonded cysteine pairs, which is encoded by avirulence gene *AvrLm4-7* from *Leptosphaeria maculans* can translocate into plant cells and interact with an unidentified cognate resistance (R) protein (Blondeau et al., 2015). In this study, most of the MbCFEM proteins contains eight conserved cysteines except MbCFEM4, MbCFEM10 and MbCFEM11, which lack two cysteines respectively (**Figure 1B**). The intramolecular disulfide bonds are considered as a key structure for the protein to stand up to the degradation from the apoplast when delivered to plant cells during infection (Kamoun, 2006). For example, any substitutions to the disulfide bonds in VdSCP76 and VdSCP77 would destroy their suppression of host immunity (Wang et al., 2022a). Disulfide bonds of several effectors including Avr2, Avr4, and Avr9 from *Cladosporium fulvum* and SsSSVP1 from *Sclerotinia sclerotiorum* were also proved to be important for protein function during the interaction between plants and pathogens (van den Hooven et al., 2001; Burg et al., 2003; Van'T Klooster et al., 2011; Lyu et al., 2016). Disulfide bonds of MbCFEM4, MbCFEM10 and MbCFEM11 may fold incorrectly because of the lack of cysteines. And as a considered candidate effector, the protein model of MbCFEM10 presented relatively low similarity with Csa2 and possessed only 3 α-helices, which may imply a weak effector function (**Figure 2**). Additionally, we found the aspartic residue was relatively conserved in CFEM domains of eight MbCFEMs (**Figure 1B**). There have been several iron uptake systems discovered in fungi and the CFEM domains in *C. albicans* have also been proved to possess a Ferric ion acquisition activity due to some axial ligands such as aspartic residue, tyrosine residue, and histidine residue (Kuznets et al., 2014). The regulation of Fe nutrition, an essential element for virulence, can enhance infection in some conditions (Albarouki and Deising, 2013; Nairz and Weiss, 2020). For example, the necrotrophic growth of *C. graminicola* on the host leaf can be suppressed when the leaf is Fe-sufficient (Ye et al., 2014). Iron robbery during the infection is important for host colonization by *Verticillium dahlia* (Wang et al., 2022a). MbCFEM proteins may also possess a possible iron acquisition function and contribute to the life history of *M. brunnea*.

Due to the lack of a trans-membrane region, a total of six MbCFEMs (MbCFEM1, 2, 6, 8, 9, and 10) were finally selected as candidate effectors. The *M. brunnea* strain J4 (isolated from *Populus* × *canadensis* Moench) was used as a template. Only two (MbCFEM6 and MbCFEM8) of them were identically amplified compared with the sequenced strain MB_m1 (isolated from NL895), respectively, while MbCFEM1 and MbCFEM9 displayed some deletions/insertions and mutations (**Supplementary Table S2**), which indicates that there is genetic diversity between different physiological races, although they are both *M. brunnea* f. sp. *monogermtubi* (MULT). The discrepancy of the ORFs was probably because these candidate MbCFEM effectors have highly similar paralogous genes or a mismatched genome assembly. It also implies that there may be some more complex mechanism in the reaction between *M. brunnea* and *Populus*.

All the cloned candidate effectors were then verified the high expression during the infection (**Figure 3**), which strongly suggested that they are effectors of *M. brunnea* f. sp. *Multigermtubi*. Secretion function of these four candidate MbCFEM effectors (**Figure 4**) and their promotion to *F. proliferatum* infection were subsequently proved, while the MbCFEM8 showed only limited facilitation (**Figure 5**). MbCFEM8 also presented relatively low expression during the infection (**Figure 3**) and cannot suppress programmed cell death (PCD) triggered by INF1 (**Figure 6**). Not all the MbCFEM proteins play a role in fungal virulence. Three GPI-anchored CFEM proteins in *Aspergillus fumigatus* were found to function on cell wall stability but no facilitation to infection (Vaknin et al., 2014). None of the CFEM containing G-protein coupled receptor in *Fusarium graminearum* is essential for infection (Jiang et al., 2019). For MbCFEM8, there may be some correlation between its inability to suppress PCD and its relatively weak function on infection promotion.

To interfere with the plant immunity pathway, fungal effectors usually have special subcellular localization according to their function. CFEM proteins are mostly located on the outer layer of the cell membrane as demonstrated in Pth11 and WISH of *M. oryzae* (Dezwaan et al., 1999; Kou et al., 2016; Nazmiara and Subhankar, 2017). Glycosylphosphatidylinositol (GPI)-anchored protein is a family of plasma membrane-anchored proteins, which is significant for the whole life history of multiple eukaryotic cells (Oliveira Garcia and Deising, 2016; Mei et al., 2022). Quite a lot of GPI-modified wall proteins of yeast were found important to osmotic stability and viability (Teparić et al., 2004). For example, GPI-anchored membrane proteins DCW1 and DFG5 were proved indispensable for cell growth in *Saccharomyces cerevisiae* (Wu et al., 2002). Several CFEM proteins have also been proved to be GPI-anchored proteins (Vaknin et al., 2014; Zhu et al., 2017). In our study, each of the four tested MbCFEM candidate effectors possesses membrane localization (**Figure 7**) and was bioinformatic predicted to possess the GPI anchor on the C-terminus (**Table 1**), suggesting that they may be potential GPI-anchored proteins.

Interestingly, we noted that MbCFEM6 partially localized to the chloroplasts (**Figure 7**). Remarkably, more evidence is emerging that chloroplast plays a pivotal role in plant immunity, it is not surprising that pathogen effectors will target it as a counterstrategy in response to chloroplast immunity (CI). To date, there have been several reports of pathogen effectors localizing to the chloroplast and interacting with chloroplast-located targets to mitigate host defense (Lu and Yao, 2018; Kretschmer et al., 2020; Kachroo et al., 2021; Littlejohn et al., 2021). In fungi, the ToxA effector from the necrotrophic fungal pathogen *Pyrenophora tritici-repentis* localizes to host chloroplasts and interacts with a chloroplast protein, ToxA Binding Protein 1 (ToxABP1), in wheat and a ToxABP1 homolog in *Arabidopsis thaliana* (Thf1) (Manning et al., 2007). More recently, the SsITL effector of another necrotrophic fungal pathogen *Sclerotinia sclerotiorum* has been shown to target the calcium‐sensing receptor (CAS) in chloroplasts to inhibit host resistance (Tang et al., 2020). Several effector proteins from the obligate fungal/oomycete pathogens [*Melampsora larici*-*populina* (Petre et al., 2015), *Puccinia striiformis* f. sp. *tritici* (Petre et al., 2016)*, Puccinia graminis* f. sp. *tritici* (Sperschneider et al., 2017), *Plasmopara halstedi* (Pecrix et al., 2019), and *Plasmopara viticola* (Liu et al., 2018)] have been predicted or experimentally shown to localize to the chloroplast, although knowledge of their host targets is limited. To date, chloroplast-targeted effectors have not been readily identified in hemibiotrophic fungus. MbCFEM6 effector identified in *M. brunnea* may be the first chloroplast-targeted effector in hemibiotrophic fungus.

In conclusion, MbCFEM1, MbCFEM6, MbCFEM8, and MbCFEM9 were ultimately identified as *M. brunnea* CFEM effectors, which act as suppressors of PCD to manipulate immunity in plants. The study puts insight into the CFEM family of *M. brunnea* and lays a foundation for the research on the interaction between *M. brunnea* and its host, which gives an impetus to explore more strategies for disease control. We would further probe the virulence contribution of MbCFEM effectors during *M. brunnea* infection of its host poplars and the molecular mechanism of MbCFEM effectors manipulating plant immunity.

## Data availability statement

The datasets presented in this study can be found in online repositories. The names of the repository/repositories and accession number(s) can be found in the article/**Supplementary Material**.

## Author contributions

Conceived and designed the experiments: YQ and QX. Performed the experiments: YQ, XinZ, and XW. Analyzed the experiment data: YQ, XinZ, and XW. Contributed reagents/materials/analysis tools: JY, XiaZ, QRZ, JL, and QZ. Wrote the paper: YQ and QX. All authors have read and approve the final manuscript.

## Funding

This work was supported, in part, by grants provided to QX by the National Natural Science Foundation of China (31600512), China Postdoctoral Science Foundation (2021M691605), and Postdoctoral Science Foundation of Jiangsu Province (2021K641C); to XiaZ, QRZ, JL by Students Practice Innovation and Training Program of Nanjing Forestry University (numbers 202210298208H and 2022NFUSPITP0364, respectively); and by the Priority Academic Program Development of Jiangsu Higher Education Institutions (PAPD).

## Acknowledgments

The authors would like to thank Prof. Qiang Cheng from Nanjing Forestry University for kindly sharing lab equipment and Dr. Yao Zhao from Nanjing Agricultural University for the plasmids offering. We also thank Justin Waletich (University of Florida) and Kai Tao (Oregon Health & Science University) for their editorial assistance.

## Conflict of interest

The authors declare that the research was conducted in the absence of any commercial or financial relationships that could be construed as a potential conflict of interest.

## Publisher’s note

All claims expressed in this article are solely those of the authors and do not necessarily represent those of their affiliated organizations, or those of the publisher, the editors and the reviewers. Any product that may be evaluated in this article, or claim that may be made by its manufacturer, is not guaranteed or endorsed by the publisher.
